# Genetic Mapping and Transcriptomic Analysis of Sepal-Derived Seed Shape in Spinach

**DOI:** 10.3390/ijms262210838

**Published:** 2025-11-07

**Authors:** Mahpara Fatima, Xiaokai Ma, Ehsan Khalid, Ray Ming

**Affiliations:** 1 College of Life Science, Center for Genomics and Biotechnology, Fujian Provincial Key Laboratory of Haixia Applied Plant Systems Biology, Fujian Agriculture and Forestry University, Fuzhou 350002, China; 2 College of Agriculture, Fujian Agriculture and Forestry University, Fuzhou 350002, China

**Keywords:** genetic mapping, seed shape, sepal morphology, spinach, transcriptome

## Abstract

Spinach is a beloved vegetable crop and widely cultivated worldwide. It is dioecious with male and female plants, although monoecious mutations exist. Female spinach exhibits two distinct sepal morphologies—thorn-shaped and round-shaped determine seed shape and strongly influence seed handling, mechanized sowing, and cultivar classification. To dissect the genetic basis of this trait, we developed an F_2_ population from contrasting parental lines and constructed a high-density linkage map with ~1615 bin markers spanning ~994.04 cm. A major 4.31 Mb genomic interval on chromosome 4, flanked by tightly linked markers, was consistently associated with sepal morphology. Transcriptome profiling across early and mature sepal developmental stages revealed significant enrichment of cell cycle-related pathways, including DNA replication, repair, mitosis, and cytokinesis. By integrating differential expression analysis with weighted gene co-expression network analysis, we identified 25 DEGs within the mapped interval, 11 of which showed strong co-expression with hub genes in trait-associated modules. Structural variation analysis further uncovered promoter and coding sequence polymorphisms in a subset of candidate genes. This study highlights 11 promising candidate genes potentially regulating sepal-derived seed morphology in spinach, rather than confirming definitive causal genes, providing valuable targets for functional validation and new insights into the genetic regulation of sepal development.

## 1. Introduction

Spinach (*Spinacia oleracea* L.) is a globally cultivated leafy vegetable of high commercial value, renowned for its rich nutritional profile, including high levels of iron, calcium, folate, and vitamins A, C, E, and K [1]. In addition to its nutritional importance, spinach serves as a model for investigating morphological variations in reproductive traits, particularly seed shape, which carries both agronomic and evolutionary relevance. Spinach cultivars can be broadly categorized into two seed morphotypes: thorny (spiny) with two to three spines and round (spineless). These morphotypes display distinct geographical patterns, with Asian accessions predominantly producing thorny seeds, and European cultivars more often exhibiting round seeds [2,3]. From an agronomic perspective, round seeds are preferred because of their uniformity and advantages in mechanized sowing, ease of seed cleaning, and reduced damage during handling and fungicide treatment [4]. Nevertheless, spiny seeds persist in breeding programs, possibly due to genetic heterozygosity. In wild accessions, thorny seeds have ecological benefit such as enhanced dispersal through adhesion to animal fur, though empirical evidence remains limited. Beyond ecological and agronomic considerations, seed morphology also serves as a key criterion for varietal identification and purity assessment, both of which are critical for spinach germplasm management and commercial seed production.

Unlike in many angiosperms, where seed shape is determined by the ovule or fruit pericarp, seed morphology in spinach is governed by the calyx-specifically, the persistent, post-anthesis sepals of the female flower. These sepals fuse and continue to grow after fertilization, encasing and protecting the developing seed [5]. The ultimate external form of the seed is thus shaped by the morphological characteristics of the accrescent sepal structure [6,7]. Comparative morphological studies have confirmed that while macro-morphological distinctions between seed types may appear subtle under stereomicroscopy, micro-morphological features, particularly those of the seed coat (in reality, the fused sepals), display significant variation. Scanning electron microscopy (SEM) revealed clear differences in surface reticulation patterns, depth and angle of interspaces, and ornamentation between thorn and round seed types [3]. Such micro-features are valuable taxonomic tools, offering a refined method for distinguishing spinach varieties at the seed level.

Persistent sepals in spinach represent a notable example of floral organ modification with functions extending beyond bud protection [8]. Across angiosperms, sepals exhibit diverse morphologies—including spiny, winged, inflated, or fleshy forms—that contribute to seed and fruit protection, photosynthesis, and dispersal strategies [9,10,11,12]. For instance, the inflated calyx of Physalis (Inflated Calyx Syndrome, ICS) regulates microclimate and enhances seed dispersal, while pappus structures in Asteraceae improve wind dispersal efficiency [13,14]. In spinach, variation in sepal growth gives rise to thorny and round seed forms, providing a unique system to study the genetic and developmental mechanisms underlying adaptive floral transformations.

The advent of next-generation sequencing (NGS), including both whole-genome resequencing and RNA sequencing (RNA-seq), has dramatically advanced this field by enabling rapid, cost-efficient detection of genetic variants and simultaneous monitoring of gene expression [15]. Whole-genome resequencing provides high-resolution datasets of genomic variation, such as single nucleotide polymorphisms (SNPs), insertions and deletions (InDels), and structural rearrangements, facilitating precise linkage between genotype and phenotype. In parallel, RNA-seq allows genome-wide assessment of transcript levels, identification of genes with differential expression, and exploration of regulatory pathways controlling trait variation, thus revealing the regulatory mechanisms underlying phenotypic diversity [15,16].

The objective of this study was to elucidate the genetic basis of sepal-derived seed shape, a qualitative and developmentally significant trait in spinach, through an integrative genomics approach. Specifically, we aimed to define the genomic locus associated with sepal morphology using a high-density genetic linkage map of an F_2_ population, and to identify candidate genes through differential expression and co-expression network analyses. This integrative approach provides a robust framework for linking phenotypic variation to underlying genetic mechanisms, enabling future functional characterization, supporting molecular breeding for improved spinach seed traits, and offering insights into the evolutionary diversification of floral organs in angiosperms.

## 2. Results

### 2.1. Genetic Inheritance of Sepal Shape in Spinach

The F_2_ population was derived from a cross between two spinach parental varieties segregated for sepal morphology. II9A0012 with thorn-shaped sepals and II9A0075 with round-shaped sepals. All F_1_ plants exhibited thorn-shaped sepals, indicating that the thorn phenotype is dominant. The segregation of sepal shape was examined in 89 F_2_ plants, among which 69 exhibited thorn-shaped sepals and 20 had round-shaped sepals. A χ^2^ test confirmed that this segregation ratio fits the expected Mendelian 3:1 ratio for a single dominant gene (χ^2^_₍3:1₎_ = 0.30 < χ^2^_0.05_ = 3.841). This result indicates that sepal morphology in this cross is controlled by a single dominant locus.

### 2.2. Whole-Genome Resequencing and Variant Calling in the F_2_ Population

To identify SNPs segregating between the two parental accessions, “II9A0012” (thorn-shaped sepals) and “II9A0075” (round-shaped sepals), whole-genome resequencing was performed. A total of approximately 480 Gb of Illumina paired-end reads were generated from the two parents and 89 F_2_ individuals. The sequencing quality was high, with over 95% of bases achieving Q30. On average, each F_2_ individual was sequenced at ~5× depth, with a mean mapping rate of 93.17% to the *Spinacia oleracea* YY reference genome. A total of 2,125,003 raw SNPs were initially detected across the mapping population. After filtering significant segregation distortion, a total of 535,592 SNPs were retained for downstream genetic map construction (Table 1).

### 2.3. Construction of Genetic Linkage Map

**Figure 1:**
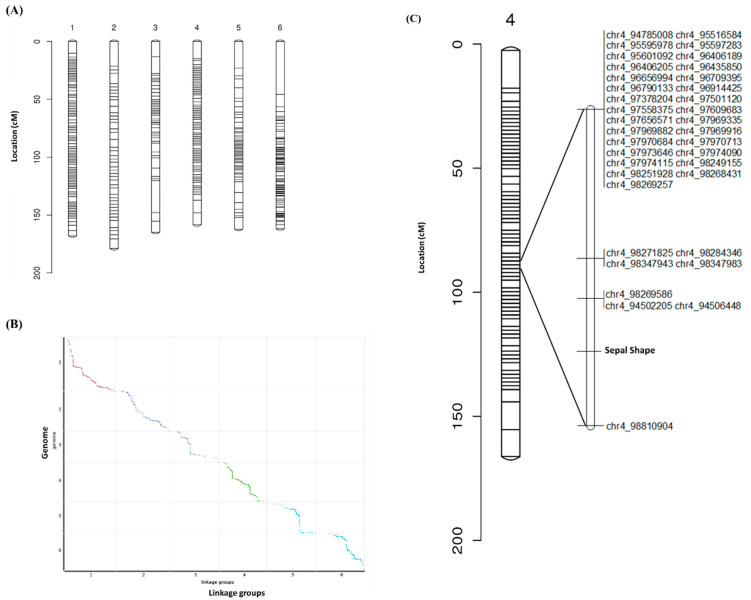
Genetic mapping of sepal morphology in spinach. (**A**) Bin map of the genetic map constructed from the “II9A0012” × “II9A0075” F_2_ population; (**B**) Comparison of Hi-C assembly and genetic map alignment. Dot plot showing correspondence between the genetic map and six pseudo-molecules of the YY spinach genome, where the horizontal axis represents genetic distance of each linkage group and the vertical axis represents genomic position; (**C**) Distribution of 37 bin markers co-segregating with sepal morphology.

215,146 markers with (aa × bb) segregation patterns were filtered into 1615 co-segregating bin markers that were mapped to six linkage groups (LGs), corresponding to the six chromosomes of spinach, and spanning a total length of 994.04 cm, with an average genetic distance of 0.62 cm between adjacent markers. Number of bin markers per chromosome ranged from 107 (chr3) to 676 (chr4) (Table 2 and Figure 1A). The largest single gap was observed on LG6 (45.80 cm), and 33 intervals across the entire map exceeded 5 cm (Table 2). A high degree of collinearity was observed between the constructed genetic map and the Hi-C–based physical assembly, indicating strong consistency between genetic and physical positions (Figure 1B).

### 2.4. Mapping of Sepal Morphology Trait

The sepal shape, a qualitative trait segregating in the F_2_ population, was included in the linkage analysis and assigned to linkage group 4 (LG4) using JoinMap^®^ 5 (Kyazma B.V., Wageningen, The Netherlands). The trait co-segregated with a cluster of 37 bin markers, and the flanking markers, chr4_94,502,205 and chr4_98,810,904, define a 4.31 Mb physical interval on the YY reference genome containing 71 annotated genes (Figure 1C and Supplementary Table S1). Linkage analysis indicated very strong association between the morphological marker and its flanking markers, with a calculated mean LOD score of 15.07, reflecting complete co-segregation in the mapping population. No recombination events were observed between the sepal shape locus and the nearest markers, confirming tight linkage. This 4.31 Mb interval represents a defined candidate region for the sepal shape locus, providing a foundation for fine mapping, structural variation analysis, and functional characterization of candidate genes.

### 2.5. Candidate Gene Identification and Expression Analysis Within the 4.31 Mb Interval

Within the 4.31 Mb genomic intervals defined by the flanking markers chr4_94,502,205 and chr4_98,810,904, a total of 71 putative genes were identified using the spinach YY reference genome. Functional annotation based on “Basic Local Alignment Search Tool Protein” (Blastp) searches against the NCBI NR database “https://blast.ncbi.nlm.nih.gov/Blast.cgi (accessed on 20 August 2022)” and Gene Ontology (GO) enrichment analysis revealed that several of these genes are involved in metabolic process, reproduction, cell cycle checkpoints, and DNA damage checkpoints.

To pinpoint candidate genes underlying sepal shape variation, transcriptome data from thorn- and round-sepal types were analyzed using a relaxed differential expression threshold of |log_2_FC| > 0.6 (approximately 1.5-fold change) and a false discovery rate (FDR) of *p* < 0.05. This adjustment was made because the number of differentially expressed genes within the genomic interval was limited under the conventional |log_2_FC| > 1 cutoff. Of the 71 genes, 26 showed no detectable expression in sepal tissues, while 18 were expressed but did not exhibit significant differential expression at any developmental stage. The remaining 27 genes were differentially expressed: 14 showed stage-specific differences at the mature stage, 4 were specific to the early stage, and 9 displayed differential expression at both early and mature stages (Supplementary Table S1).

### 2.6. Global Transcriptome Analysis of Sepal Morphology in Spinach

**Figure 2:**
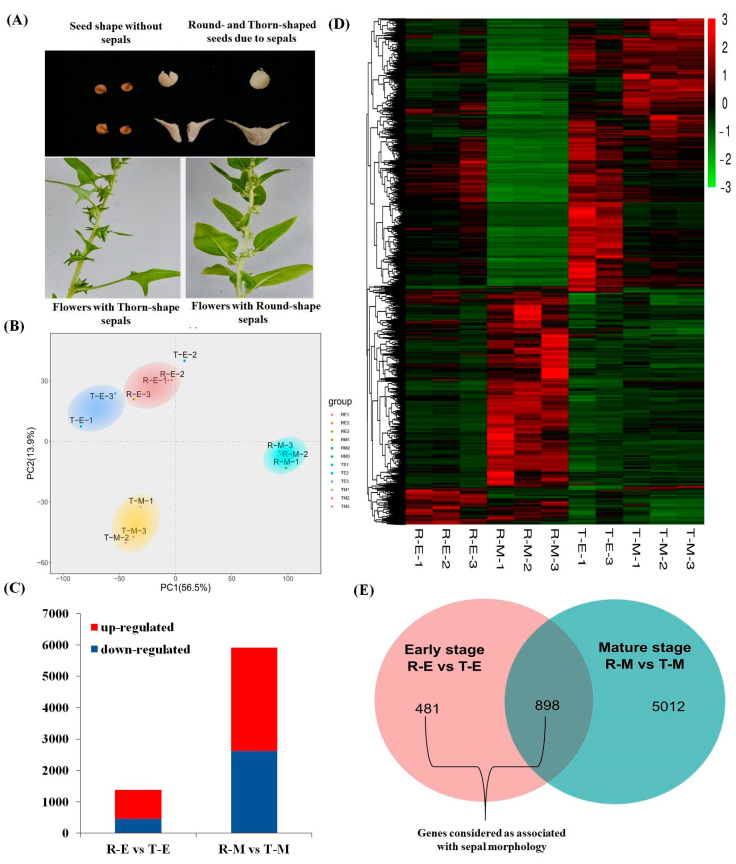
(**A**) Phenotypes of round and thorn female flower sepals; (**B**) Principal component analysis (PCA) of transcriptome samples; (**C**) Numbers of up- and down-regulated genes at early and mature stages when comparing round and thorn sepals; (**D**) Heatmap of gene expression profiles across four sample types, each with three biological replicates (note: sample T-E-2 was removed); (**E**) Venn diagram showing DEGs shared between early and mature stages (R-E vs. T-E and R-M vs. T-M).

To investigate transcriptomic changes associated with sepal shape, RNA-seq analysis was performed on four sample groups representing round (R) and thorn (T) sepal tissues at two developmental stages: early (E) and mature (M). Following quality control, 1.5–2.5% of raw reads were removed, resulting in 96.6–97.6% of clean reads mapping to the YY reference genome, with uniquely mapped reads ranging from 92.4% to 93.4%, and multi-mapped reads between 3.7% and 4.9%. Transcript abundance was quantified as fragments per kilobase of transcript per million mapped reads (FPKM) for each gene in all samples. Principal component analysis (PCA) conducted using the DESeq2 R package (1.44.0) identified outlier libraries, while pairwise scatterplots demonstrated strong correlations (r^2^ > 0.8) among biological replicates, with the exception of one early-stage thorn replicate (T-E-2), which, despite adequate sequencing depth, failed to cluster with its replicates (Figure 2A). To improve analytical robustness, this outlier was excluded from downstream analyses.

Differential expression patterns were visualized using a heatmap generated by the ‘pheatmap’ R package (1.0.12), which confirmed improved consistency among retained replicates (Figure 2C). Comparative analysis identified 481 early-stage biased differentially expressed genes (DEGs) and 5012 mature-stage biased DEGs between thorn and round sepal types. Additionally, 898 DEGs exhibited differential expression at both early and mature stages. These DEGs were selected based on thresholds of |log_2_ (FPKM_T/FPKM_R)| > 1 or < −1, *p* < 0.05, and false discovery rate (FDR) ≤ 0.05 (Figure 2B,D).

### 2.7. Functional Annotation and Expression Analysis of Early-Stage DEGs Associated with Sepal Morphology

As the sepal represents the first and outermost floral organ to initiate development in spinach, its morphological identity is likely determined during the early stages of floral ontogeny. To investigate the molecular mechanisms underlying sepal shape differentiation, Gene Ontology (GO) enrichment analysis was conducted on a set of 1379 differentially expressed genes (DEGs), comprising 481 early-stage-biased DEGs and 898 DEGs shared across both early and mature stages. Enrichment was assessed across three main GO categories—biological process, cellular component, and molecular function—using a significance threshold of −log_10_ (*p*-value) > 1.3 (*p* < 0.05).

**Figure 3:**
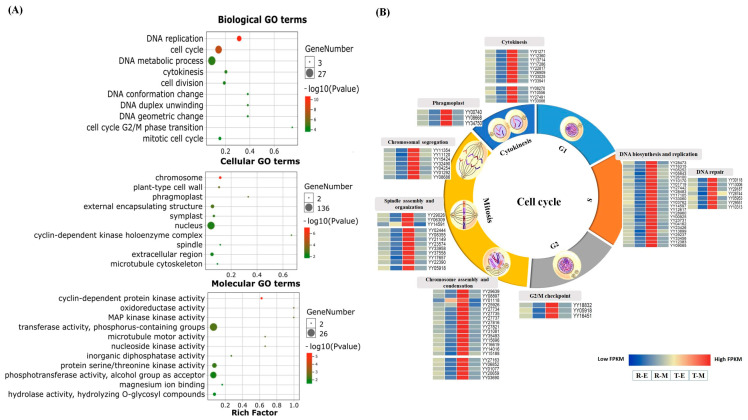
(**A**) Top enriched GO terms of 1379 differentially expressed genes (DEGs) associated with sepal morphology; (**B**) Expression patterns of cell cycle-related genes in round (R) and thorn (T) sepal samples at early (E) and mature (M) developmental stages.

Among the biological processes, highly overrepresented terms included DNA replication, DNA metabolic process, cytokinesis, cell division, DNA confirmation change, duplex unwinding, and cell cycle G2/M phase transition pointing to robust regulation of the cell cycle. Within the cellular component category, the most enriched terms were chromosome, plant-type cell wall, phragmoplast, nucleus, cyclin-dependent kinase holoenzyme complex, spindle, and microtubule cytoskeleton-related components. Enrichment in the molecular function category highlighted cyclin-dependent protein kinase activity, oxidoreductase activity, MAP kinase activity, microtubule motor activity, nucleoside kinase activity, inorganic diphosphatase activity, phosphotransferase activity, and hydrolase activity (Figure 3A).

Furthermore, expression profiling of genes annotated to the cell cycle pathway revealed significantly higher expression levels in thorn-shaped sepals compared to round-shaped ones, particularly during the S-phase (DNA replication), M-phase (chromosome condensation, spindle assembly, and chromosome segregation), and cytokinesis. This suggests that modulation of the cell cycle is strongly associated with sepal morphology variation in spinach flowers (Figure 3B, Supplementary Table S2).

### 2.8. Integration of Candidate Genomic Region with Co-Expression Networks Reveals Putative Regulators of Sepal Morphology

To identify key regulators of sepal morphology, we integrated gene co-expression network analysis with a candidate genomic region defined by linkage mapping. In parallel, we constructed a weighted gene co-expression network using 1450 genes (including 1379 differentially expressed genes from the early and mature developmental stages, and 71 genes within the mapped interval) using WGCNA in R. Genes exclusively expressed at the mature stage were excluded to minimize background noise, given that sepal shape determination occurs during early development. A weighted gene co-expression network was constructed with a soft threshold power of 7, satisfying the criteria for scale-free topology and mean connectivity. All samples passed outlier detection using the “ward.D2” hierarchical clustering method. The expression data were subsequently decomposed into five subnetwork modules. The turquoise module was the largest, containing 1120 genes, followed by blue (193 genes), brown (180 genes), and gray, the smallest, with only seven genes.

**Figure 4:**
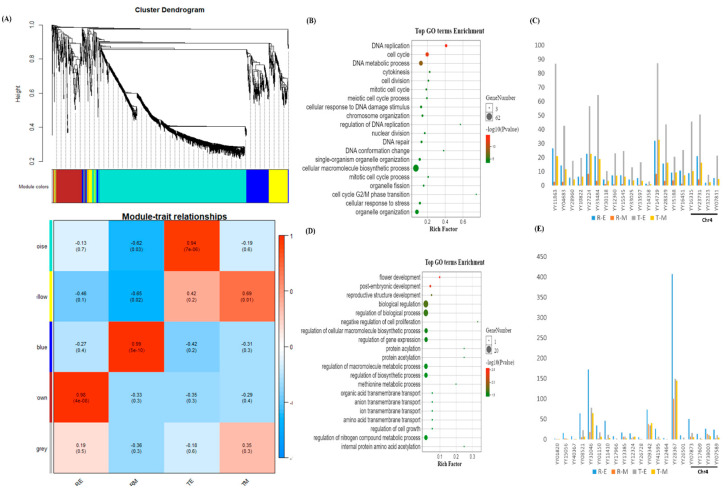
(**A**) Module–trait relationships for five modules. Each row represents a module defined as the first principal component of the gene expression profiles within that module, with module eigengene expression considered as a representative of all genes. Each column corresponds to a trait. Numbers within cells indicate correlation coefficients with associated *p*-values (in brackets). The table is color-coded according to correlation strength (see legend). RE, round sepal at early stage; RM, round sepal at mature stage; TE, thorn sepal at early stage; TM, thorn sepal at mature stage; (**B**) Top enriched GO terms of the turquoise module; (**C**) Expression profiles of the top 20 hub genes in the turquoise module; (**D**) Top enriched GO terms of the brown module. (**E**) Expression profiles of the top 20 hub genes in the brown module.

Because sepal morphology is developmentally determined at an early stage, a module–trait correlation analysis showed two contrasting modules — turquoise and brown—with significant correlations to sepal shape at the early stage. The turquoise module showed a strong positive correlation with thorn-shaped sepals (r = 0.94, *p* < 7 × 10^−6^) and was therefore considered a hub module (Figure 4A). Gene ontology analysis revealed enrichment in pathways related to DNA replication, cell cycle, cell division, and macromolecule biosynthesis (Figure 4B). Twenty candidate hub genes from this module were identified based on high intra-modular connectivity and edge degree, showing strong expression in thorn-shaped morphologies (Figure 4C, Supplementary Table S3)

Conversely, the brown module showed a strong positive correlation with round-shaped sepals (r = 0.98, *p* < 4 × 10^−8^) and was negatively correlated with the turquoise and yellow modules (Figure 4A). Genes in this module were enriched for functions related to regulation of biological processes, transcriptional regulation, and negative regulation of cell proliferation (Figure 4D). Twenty hub genes were identified, suggesting potential regulatory roles in round sepal morphology (Figure 4E, Supplementary Table S3).

Mapping the hub genes from both modules to the candidate region revealed that while no hub gene directly overlapped with the genomic interval (94,502,205 bp to 98,810,904 bp), four turquoise module hub genes—YY16315 (RNR2-TSO2), YY23731 (flap endonuclease 1 isoform X3), YY32323 (DNA cross-link repair 1 protein-like), and YY07811 (GDSL esterase/lipase *At4g16230*-like)—and four brown module hub genes—YY07873 (mitochondrial import inner membrane translocase subunit Tim17), YY38003 (probable serine/threonine-protein kinase PIX13), YY07589 (cuticle collagen 34-like), *and YY17609* (late embryogenesis abundant protein D-34-like)—were located on chromosome 4 (Supplementary Table S3).

**Figure 5:**
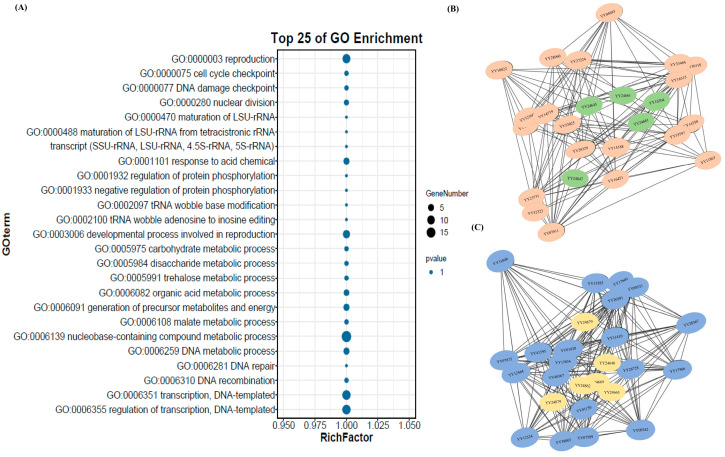
(**A**) Top enriched GO terms for 71 genes within the candidate region associated with sepal shape; (**B**) Co-expression network showing candidate genes (green circles) with the top 20 hub genes in the turquoise module; (**C**) Co-expression network showing candidate genes (yellow circles) with the top 20 hub genes in the brown module.

**Figure 6:**
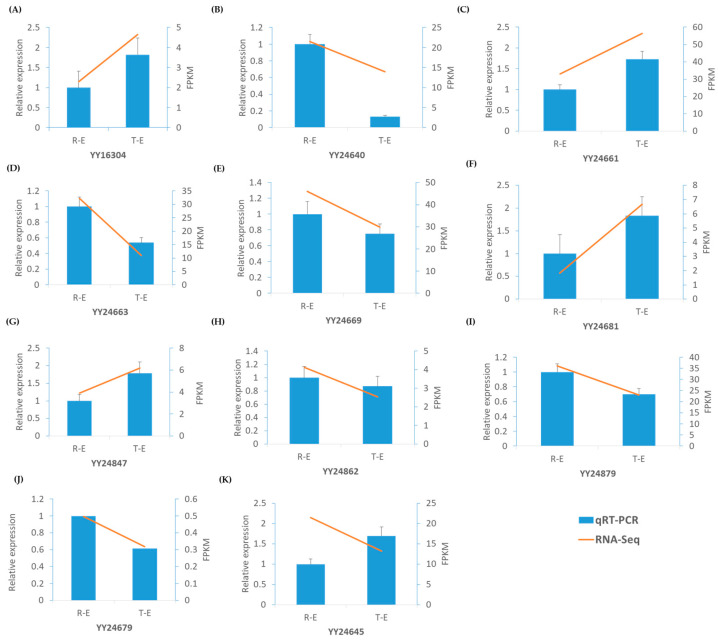
qRT-PCR analysis of eleven candidate genes during early sepal development in spinach. Relative expression levels were compared between round (RE) and thorn (TE) sepals. (**A**) *YY16304* showed low expression in RE than TE; (**B**) *YY24640* was higher in RE than TE; (**C**) *YY24661* increased markedly in TE; (**D**) *YY24663* decreased from RE to TE; (**E**) *YY24669* was slightly higher in RE and reduced in TE; (**F**) *YY24681* was higher in TE; (**G**) *YY24847* was low in RE but strongly increased in TE; (**H**) *YY24862* was moderately higher in RE and slightly decreased in TE; (**I**) *YY24879* decreased from RE to TE; (**J**) *YY24679* was slightly higher in TE; (**K**) *YY24645* increased in TE relative to RE. The qRT-PCR results showed distinct expression patterns between round and thorn sepals, consistent with RNA-Seq data, confirming the reliability of transcriptomic. Error bars represent the standard error of the mean (SEM) from three biological replicates.

Cross-referencing the candidate genomic region genes with the co-expression modules showed that 15 genes from the candidate region were co-expressed within the turquoise module and nine in the brown module (Supplementary Table S1). Among these, five genes in the turquoise module exhibited high connectivity (weight > 0.5) with hub genes, including YY24645 (transcription factor HHO2-like isoform X1), YY24661 (plasmodemata callose-binding protein 1-like), YY24681 (homologous-pairing protein 2 homolog), YY16304 (probable NOT transcription complex subunit VIP2 isoform X1), YY24847 (piriformospora indica-insensitive protein 2-like isoform X1). In the brown module, six genes—YY24669 (ATP-dependent Clp protease adapter protein CLPS1) and YY24879 (coiled-coil domain-containing protein 130), and YY24679 (E3 ubiquitin-protein ligase SP1 isoform X1), YY24862 (WD-40 repeat family protein), YY24640 (AP2/ERF and B3 domain-containing transcription factor RAV1-like), YY24663 (uncharacterized protein) —displayed similarly high connectivity with corresponding module hub genes. Importantly, all 11 genes displayed differential expression at early or early-mature stages between contrasting sepal phenotypes (Figure 5B,C). Among these, seven genes (YY24862, YY24645, YY24640, YY24669, YY24879, YY24663 and YY24679) showed higher expression in the round-sepal phenotype, whereas four genes (YY24661, YY24681, YY16304, YY24847) were more highly expressed in the thorn-sepal phenotype (Supplementary Table S1). Further, we examined the expression of these genes at the early stage of sepal development using qRT-PCR. Th results were consistent with the RNA-seq data, except for YY24645 (*transcription factor HHO2-like isoform X1*), which showed higher expression in thorn-type sepals than in round-type sepals (Figure 6). These findings highlight a subset of strongly co-expressed and differentially expressed genes within the candidate region that may be involved in regulating sepal morphology.

### 2.9. Structural Variation Analysis of Candidate Genes

A comprehensive summary of structural variation for all 71 genes is provided in Supplementary Table S4. Among the 11 candidate genes as describe above, several exhibited notable sequence differences between thorn- and round-sepal genotypes. YY24679 (E3 ubiquitin-protein ligase SP1 isoform X1) displayed five SNPs in genomic sequence at positions 94,706,449 (C→T), 94,706,542 (A→C), 94,706,538 (T→A), 94,705,543 (C→G), and 94,706,606 (G→C), along with a promoter SNP at 94,704,220 (C→T). YY24879 (coiled-coil domain-containing protein 130) contained three coding SNPs at positions 98,400,597 (G→A), 98,400,675 (C→T), and 98,400,704 (G→T). Promoter SNPs were observed in YY24661 (plasmodemata callose-binding protein 1-like) at 95,970,949 (T→A) and YY24847 (piriformospora indica-insensitive protein 2-like isoform X1) at positions 98,396,296 (G→T), 98,396,740 (C→T), 98,396,797 (G→T), and 98,396,374 (A→G). These variations suggest potential regulatory or functional differences in these candidate genes that may contribute to the thorn- versus round-sepal phenotypes.

## 3. Discussion

Breeders are committed to delivering high-quality spinach seeds with superior yield potential, multi-disease resistance, and desirable quality traits to support sustainable agriculture [17]. Seed morphology—particularly the presence or absence of spines is a key characteristic in cultivar classification, seed purity assessment, mechanized sowing, and natural dispersal strategies [18]. In spinach, the sepal of female flowers develops into two distinct morphologies: thorn-shaped (spiny) and round-shaped (non-spiny). These structures are evolutionarily modified sepals that encapsulate and mature with the seed, ultimately determining its final shape [6,7]. Such morphological differences are likely governed by genetic regulation of developmental pathways. To date, limited studies have investigated the genetic basis of seed shape in spinach. A notable example is the work by Liu et al. who used recombinant analysis in a BC_1_ population to localize the dominant Fs gene to a 1.9 Mb region on chromosome 3, which was subsequently narrowed to a 0.27 Mb interval using a recombinant inbred line (RIL) population [18]. Based on the Sp75 reference genome, four candidate genes were proposed for the spiny seed trait. However, the authors noted potential limitations in the reference assembly quality, acknowledging that incomplete or fragmented genome sequences within the Sp75 assembly could have resulted in missing or incorrectly annotated candidate genes. Indeed, multiple assembly gaps were present within the 0.27 Mb target region, likely contributing to the loss or mis-annotation of relevant genetic elements [18,19].

In the present study, we utilized the high-quality YY reference genome, thereby enhancing sequence continuity and gene annotation accuracy [20]. By integrating high-density genetic mapping with transcriptome profiling, we not only delineated the candidate genomic region but also uncovered functional insights into the regulatory networks underlying sepal-derived seed shape. Unlike the previous report, our genetic mapping of an F_2_ population derived from contrasting sepal types—II9A0012 (thorn-shaped sepals) and II9A0075 (round-shaped sepals)—localized the sepal shape locus to a 4.31 Mb interval on chromosome 4, flanked by markers chr4_94,502,205 and chr4_98,810,904, and encompassing 71 annotated genes.

To further elucidate the regulatory basis of sepal shape variation in spinach, we analyzed transcriptome dynamics between parental genotypes at both early and mature stages of sepal development. This comparative analysis revealed a significant enrichment of cell cycle-related pathways, with genes involved in S-phase progression, G_2_/M transition, chromosome organization, and cytokinesis showing significant expression divergence between contrasting sepal types from the earliest developmental stage. The central role of cell division in shaping plant organ morphology is well established: variation in the rate, orientation, and timing of cell proliferation directly influences organ size, shape, and tissue architecture [21]. Cell division rate, orientation, and growth patterns critically shape organ morphology, as shown in cucumber, tomato, and other species where altered cell cycle regulation drives fruit, leaf or sepal form [22,23,24,25]. Taken together, these findings indicate that differential regulation of cell cycle-related pathways provides a plausible mechanistic basis for the observed variation in sepal-derived seed shape in spinach.

In this study, 13 of the 71 genes within the 4.31 Mb candidate intervals were differentially expressed between thorn- and round-shaped sepals at early and early-to-mature developmental stages. Eleven of these genes were co-expressed with hub genes in key WGCNA modules, suggesting potential regulatory roles in sepal morphology. Four genes (YY24679, YY24879, YY24661, and YY24847) exhibited structural variation. YY24679 is orthologous to the mitochondrial outer membrane E3 ubiquitin ligase SP1 in plants, which remodels organelle proteomes by targeting translocon components for degradation in response to developmental and stress signals [26]. YY24879, a coiled-coil protein, is essential for spliceosome maturation and cell cycle regulation in humans [27]. In plants, coiled-coil proteins mediate signaling and development, such as N’-THF1 interactions that regulate light-dependent cell death and stress responses [28]. YY24661, a plasmodesmata callose-binding protein (PDCB), ortholog regulates intercellular communication in plants by modulating callose deposition at plasmodesmata, thereby influencing tissue patterning [29]. YY24847, encoding a Piriformospora indica-insensitive protein 2 (PII-2)–like protein, contributes to plant growth and development; in Arabidopsis, PII-2, a leucine-rich repeat protein at the ER–plasma membrane interface, promotes growth and seed production in association with the endophytic fungus P. indica [30]. Among the six hub-associated candidate genes, YY24645, and HHO2-like transcription factor, coordinates flowering time in Arabidopsis through PRR9, PRR7, and PRR5 by promoting CONSTANS expression and downstream photoperiodic genes, ensuring proper developmental timing [31]. YY24669, encoding an ATP-dependent Clp protease adapter protein, maintains organelle proteostasis by degrading misfolded or damaged proteins in chloroplasts and mitochondria, indirectly influencing development and tissue organization [32]. YY24681, a homologous-pairing protein 2 (HOP2) homolog, ensures accurate homologous chromosome pairing and recombination during meiosis, supporting genome stability and normal organ development [33]. YY16304, encoding transcription complex subunit VIP2, regulates genes involved in Agrobacterium-mediated transformation and abiotic stress responses in Arabidopsis, acting as a transcriptional modulator of stress signaling and transformation efficiency [34]. YY24663 was defined as uncharacterized gene with significant difference in expression between sepal types.

Interestingly, YY24862 encodes a WD-40 repeat family protein that serves as a scaffold for multi-protein complexes, regulating chromatin assembly, signal transduction, and cell cycle progression. In Arabidopsis, the WD-40 protein MSI1 is essential for S-phase progression, seed development, and floral organ formation [35]. YY24640 encodes an AP2/ERF and B3 domain-containing transcription factor RAV1-like, which integrates cytokinin signaling to control root meristem size and cell proliferation, linking it to cell cycle regulation [36]. Additionally, RAV1 acts as a negative regulator of seed and organ development, influencing overall organ morphology and growth [37].

Although several candidate genes with structural variations were identified, genes lacking sequence changes cannot be overlooked. Their differential expression and co-expression with hub genes suggest that these genes may influence sepal morphology through epigenetic modifications, or post-translational mechanisms, rather than through direct sequence alterations. Previous studies have shown that genes without structural polymorphisms can still modulate developmental processes by altering expression levels, protein stability, or interactions with other regulatory components [38]. Therefore, both structural and non-structural variants are likely to contribute to the genetic regulation of organ shape.

In this study, the relatively small F_2_ population (*n* = 89) may limit the resolution of linkage mapping and the detection of rare recombination events. Small populations can lead to overestimation of effect sizes and reduce the power to detect minor-effect loci, potentially biasing candidate gene identification. Despite these limitations, the high-density SNP map and strong co-segregation observed for the sepal shape locus indicate that major-effect genes were reliably captured. Future studies employing larger F_2_ populations (e.g., 200–500 individuals) or advanced mapping populations such as recombinant inbred lines (RILs) could improve mapping precision. RILs are particularly advantageous because they accumulate multiple generations of recombination and achieve near-homozygosity, thereby increasing mapping resolution and allowing repeated phenotyping across environments. Similar strategies have been successfully used in rice, soybean, and Brassica for fine mapping of developmental and morphological traits [39,40,41,42,43,44]. Nonetheless, functional characterization of these candidate genes is still required to confirm their regulatory roles.

## 4. Materials and Methods

### 4.1. Plant Material and Construction of F_2_ Population

The Chinese cultivars II9A0012 (accession with thorn seeds) and II9A0075 (accession with round seeds) were obtained from Chinese Academy of Agriculture Sciences, China, and were planted in the growth room of Haixia Institute of Science and Technology (HIST), Fujian Agriculture and Forestry University, Fuzhou, China, with temperatures set at 23 °C, humidity 65% and a 16 h photoperiod. The F_2_ mapping population was constructed by selfing of F_1_ individuals from the cross between “II9A0012” (as maternal parent, flowers with thorn-shaped sepals) and “II9A0075” (as paternal parent, female plant flowers with round-shaped sepals) (Figure 2A). Sepal phenotype of each female individual in F_2_ population was evaluated, and found to be segregated in a ratio of T (thorn):R (round) ≈ 3:1.

### 4.2. Whole-Genome Resequencing and Variant Calling

A total of 91 individuals, including 89 (69 thorn: 20 round) from an F_2_ mapping population and both parents (II9A0012 and II9A0075), were selected for whole-genome resequencing. Genomic DNA was extracted from young leaf tissue using the QIAGEN DNeasy Plant Mini Kit (QIAGEN, Hilden, Germany) “https://www.qiagen.com/ (accessed on 1 January 2022)”. Paired-end libraries (150 bp) were prepared using the NEBNext Ultra DNA Library Prep Kit Shirley, USA and sequenced on the Illumina HiSeq 2500 platform. The 89 F_2_ individuals were sequenced at approximately 5× coverage, while the maternal and paternal parents were sequenced at ~15× coverage. Raw paired-end reads were assessed for quality using FastQC “https://www.bioinformatics.babraham.ac.uk/projects/fastqc/ (accessed on 6 March 2022)” and trimmed using Trimmomatic. High-quality reads were aligned to the *Spinacia oleracea* YY reference genome using Bowtie2 with default parameters. Variant calling was performed using the Genome Analysis Toolkit (GATK) via the HaplotypeCaller module. Initially, 2,125,003 unfiltered variants (SNPs and InDels) were identified. Variant filtering retained 535,592 high-quality variants after removing those with depth (DP) < 2 or > 60, base quality (minQ) < 20, missing rate > 20%, or minor allele frequency (MAF) < 5%.

### 4.3. Genotyping and Genetic Linkage Map Construction

Genetic maps of “II9A0012 × II9A0075” population were constructed using bin markers derived from re-sequencing SNPs with the YY reference genome using MSTmap "http://mstmap.org/ (accessed on 10 December 2022)”. During variant calling, multiple segregation types were detected, including:

- aa × bb: both parents homozygous for alternate alleles; progeny segregate 1:2:1 (AA:AB:BB)—fully informative for F_2_ mapping.
- ab × cc: one parent heterozygous (AB), other homozygous (CC); progeny segregate 1:1.
- ab × cd: both parents heterozygous for different alleles; progeny segregate 1:1:1:1.
- ac × ab, ef × eg: one or both parents heterozygous with one shared allele; progeny segregate 1:1:1:1.
- hk × hk: both parents heterozygous for the same alleles; progeny segregate 1:2:1.
- lm × ll, nn × np: one parent heterozygous, other homozygous; progeny segregate 1:1.

For F_2_ mapping, only (aa × bb) markers, where the maternal parent is homozygous (aa) and the paternal parent is homozygous (bb), were selected. Other types are typically used in backcross, RIL, or outcrossing populations and were excluded from the final map [45]. Selected markers were further filtered using following criteria: offspring depth ≥ 3×; parental depth ≥ 10×; missing rate < 0.3; segregation distortion (*p* < 0.01); without abnormal bases. Finally, 1615 bin markers were used to build genetic map. The linkage groups were partitioned based on the chromosomal assembly of Hi-C physical map.

### 4.4. Mapping of Sepal Morphology Trait

The sepal shape trait was mapped onto a linkage group based on the genotypes of bin markers using JoinMap^®^ 5 (Kyazma B.V., Wageningen, and The Netherlands). Linkage groups were assigned at a minimum LOD score threshold of 4.0 using the regression mapping algorithm and Kosambi mapping function. The markers most tightly linked to the sepal shape were identified by visual inspection of recombination patterns. Flanking markers on either side of the associated locus were used to delimit a candidate genomic interval. Genes within the defined interval were extracted based on the YY genome annotation (Genome Warehouse, BioProject ID: PRJCA004899 [20].

### 4.5. RNA Extraction, Library Preparation, Global DEGs Analysis

The parental accessions—II9A0075 (round-shaped sepals) and II9A0012 (thorn-shaped sepals)—used to generate the F_2_ population were selected for RNA sequencing. Female flowers of both sepal types were collected at two developmental stages: (i) early stage (0.5–1 mm average size) and (ii) mature stage, when flowers were fully developed and ready for pollination (3–4 mm average size). Each sample consisted of three biological replicates, with 15–20 flowers per replicate. Sepal tissues were dissected under a stereomicroscope, separated from other floral parts, and washed with double-distilled water (ddH_2_O) to eliminate contaminants. All samples were frozen in liquid nitrogen and stored at −80 °C.

Total RNA was extracted using the RNeasy Plant Mini Kit (QIAGEN, Hilden, Germany) “https://www.qiagen.com/ (accessed on 5 March 2023)”. RNA concentration and integrity were assessed using agarose gel electrophoresis and the Agilent 2100 Bioanalyzer (The Lab World Group). First-strand cDNA synthesis was performed using the PrimeScript™ RT Reagent Kit (TaKaRa, Dalian, China), and sequencing libraries were prepared with the NEBNext Ultra RNA Library Prep Kit (Ipswich, Massachusetts), following the manufacturer’s instructions. Paired-end sequencing (150 bp) was performed on the Illumina HiSeq™ 2500 platform. Raw reads were quality-filtered using Trimmomatic with default parameters to remove adapters, poly-N sequences, and low-quality reads. Clean reads were mapped to the YY reference genome using the STAR aligner. Transcript assembly and FPKM (Fragments per kilo-base of transcript per Million mapped reads) quantification were conducted using StringTie. Read count tables were generated using the prepDE.py script“http://ccb.jhu.edu/software/stringtie/index.shtml?t=manual (accessed on 10 May 2023)”.

DEGs analysis was performed using the DESeq2 package in R. Genes were considered differentially expressed if |log_2_ fold change| > 1 and adjusted *p*-value ≤ 0.05. Pairwise comparisons were made between round (R) and thorn (T) sepals at each developmental stage: (i) RE vs. TE (early stage), and (ii) RM vs. TM (mature stage). Functional enrichment analysis of DEGs was conducted using OmicShare “https://www.omicshare.com/ (accessed on 7 June 2023)” and PlantRegMap [46].

### 4.6. Co-Expression Analysis and Candidate Genes Prediction

Co-expression analysis and candidate gene prediction were performed using FPKM datasets of DEGs from early and early-mature stages, combined with genes located within the candidate genomic region, in R (v4.3.1) using the WGCNA package (v1.51) [17]. The network topology was assessed using the pickSoftThreshold function to select a soft-thresholding power that satisfied the scale-free topology criterion (R^2^ > 0.8). Modules were detected using the blockwiseModules function with parameters set to unsigned TOM, mergeCutHeight = 0.25, soft power = 7, and a minimum module size of 30. Hub genes were identified within modules significantly correlated with sepal morphology based on intra-modular connectivity (KME) and edge degree, using thresholds commonly applied in plant WGCNA studies (e.g., KME > 0.8). Functional classification of candidate modules was conducted using Gene Ontology (GO) and KEGG (KO) databases via OmicShare “https://www.omicshare.com/ (accessed on 10 June 2023)” and BLASTP. For unannotated genes, conserved domains were identified using NCBI Batch CD-Search “https://www.ncbi.nlm.nih.gov/ (accessed on 15 June 2023)” and interProScan “https://www.ebi.ac.uk/interpro/ (accessed on 6 July 2023)”. Module-trait correlations were computed using Pearson correlation to relate gene expression patterns to sepal morphology.

### 4.7. Quantitative Real-Time PCR (qRT-PCR) Analysis

Quantitative real-time PCR (qRT-PCR) was performed using an ABI 7500 Fast Real-Time PCR System (Applied Biosystems, Foster City, CA, USA) with a two-step amplification protocol following the manufacturer’s instructions. The same RNA samples used for RNA-seq library construction were used for validation. First-strand cDNA was synthesized from 1 µg of total RNA using the PrimeScript™ RT Reagent Kit (TaKaRa, Dalian, China) and diluted to a final volume of 80 µL. qRT-PCR reactions were carried out in a 20 µL volume containing 1 µL of diluted cDNA, 10 µL of TB Green™ Premix Ex Taq™ II (TaKaRa), and 1 µM of each gene-specific primer. The thermal cycling conditions consisted of initial denaturation at 95 °C for 30 s, followed by 40 cycles of 95 °C for 5 s and 60 °C for 34 s and a dissociation stage as per the manufacturer’s instructions. A melting curve analysis was performed to confirm amplification specificity. Gene expression levels were calculated using the 2^−ΔΔCt^ method, with Actin serving as an internal reference for normalization [47]. All reactions were performed in three biological replicates with three technical replicates each. Primers were designed using SnapGene software “https://www.snapgene.com/ (accessed on 20 August 2025)”and are listed in Supplementary Table S5.

## 5. Conclusions

In this study, genetic mapping of spinach revealed a major locus for sepal-derived seed shape on chromosome 4. Transcriptome analysis showed differential expression of cell cycle- and division-related genes during sepal development, contributing to the formation of thorn and round seeds. We further combined genetic mapping, differential expression analysis, and co-expression network analysis, which resulted in the identification of eleven strong candidate genes. This study provides a solid foundation for identifying potential candidate genes, rather than confirming definitive causal genes. Our findings will support future fine mapping and the genetic improvement of sepal-derived seed morphology in spinach.

## Figures and Tables

**Table 1:** Statistics of different genetic markers used in genetic map.

Marker Type	SNP Number	INDEL Number	Percentage
Aaxbb	215,146	198,316	16,830
Abxcc	426	145	281
Abxcd	16	0	16
Acxab	352	112	240
Efxeg	471	184	287
Hkxhk	34,931	33,020	1911
Lmxll	152,440	142,726	9714
Nnxnp	131,810	123,140	8670
Total	535,592	497,643	37,949

**Table 2:** Statistics of genetic map of “II9A0012” × “II9A0075” F_2_ population.

LGID	Number Marker	Number Unit	Total Distance	Average Distance	Gap > 5 cm	Max Gap
1	330	93	167.87	0.51	1	10.23
2	140	44	178.92	1.29	10	21.33
3	107	42	164.83	1.55	8	27.52
4	676	72	158.39	0.23	3	14.75
5	206	51	162.20	0.79	8	23.22
6	156	76	161.83	1.04	3	45.80
total	1615	378	994.04	0.62	33	45.80

## Data Availability

Data will be submitted and made publicly available after this submission; this statement will be edited in revision.

## Supplementary Materials

The following supporting information can be downloaded at: https://www.mdpi.com/article/10.3390/ijms262210838/s1.
